# Assessing baseline knowledge and practices of injection safety among primary health care workers in Cross River State, Nigeria: a cross-sectional urban-rural comparative study

**DOI:** 10.11604/pamj.2021.38.35.17495

**Published:** 2021-01-14

**Authors:** Obaji Etaba Akpet, Nnette Okon Ekpenyong, Nkese Effiong Mkpanam, Soter Ameh, Angela Ekanem Oyo-Ita, Chikaike Ogbonna, Bassey Maundy Ikpeme

**Affiliations:** 1 Department of Community Medicine, Faculty of Medicine, College of Medical Sciences, University of Calabar, Calabar, Cross River State, Nigeria,; 2 Department of Community Medicine, Faculty of Health Sciences, College of Medical Sciences, University of Jos, Jos, Plateau State, Nigeria; 3 Department of Community Medicine, University of Calabar Teaching Hospital, Calabar, Cross River State, Nigeria

**Keywords:** Knowledge, practices, injection, safety, primary health care workers, urban, rural

## Abstract

**Introduction:**

unsafe injection practices are commonplace in low-income countries, and place health care workers at risk of blood-borne infections. A safe injection strategy requires a synchronized approach to deal with change in behavior of users and service providers towards safer practice. There is general lack of data on injection safety practices in Cross River State. This was a baseline study to compare the knowledge and practice of safe injection practices among primary health care (PHC) workers in urban and rural health facilities in Cross River State, Nigeria.

**Methods:**

this was a cross-sectional comparative study among PHC workers in randomly selected rural and urban Local Government Areas (LGAs). Using multistage sampling technique, a total of 320 respondents: 160 from the urban LGAs and 160 from the rural LGAs were interviewed. Semi-structured interviewer administered questionnaires were used to obtain data. Data analysis was done using STATATM version 14.0. Associations were tested using Chi square, and multivariate logistic regression analysis.

**Results:**

in this study, there was no difference in the baseline knowledge (58.8% vs. 55.0%, P=0.499) and practice (33.1% vs. 34.4%, P=0.813) of injection safety between PHC workers in the urban and rural locations. In the multivariate logistic regression model, the senior health workers had a two-fold increased odds of practicing safe injection compared to their junior counterparts [OR=2.21 (95% CI: 1.28,3.84)].

**Conclusion:**

in both the urban and rural locations, there was good knowledge but poor practice of injection safety among respondents in the LGAs; hence, the need to organize periodic injection safety training and retraining of PHC workers targeting junior workers to improve on the practices of injection safety.

## Introduction

In 2000, the World Health Organization (WHO) estimated that 501,000 deaths occurred because of unsafe injection practices [1]. The 2002 World Health Report (WHR) showed that unsafe injection practices accounted for 30% of Hepatitis B Virus (HBV) infections, 31% of Hepatitis C Virus (HCV) infections, 28% of liver cancer, 24% of liver cirrhosis cases, 5% of Human Immunodeficiency Virus (HIV) infections and 0.9% deaths worldwide [2]. There is an estimated global financial cost of US$535 million per year, and a calculated unsafe practice that is associated with 1.3 million annual deaths and 26 million years of life lost [3].

The WHO estimates that 12 billion injections are given annually, 5% of which are administered for immunization and 95% for curative purposes [4]. Unsafe injection practices, especially needle and syringe re-use are common place in low-income country health settings, and place health care workers, patients and the community at risk of infection with blood-borne viruses. It is estimated that up to 160,000 HIV, 4.7 million hepatitis C and 16 million hepatitis B infections each year are attributable to these practices [5]. Twelve billion injections are given each year in developing and underdeveloped countries. Seventy percent (70%) of these injections are unnecessary, and oral medications could have sufficed [6]. The problem is complex and fueled by weak funding of public health services. The consequences of unsafe injection practices lead to disability and death. The WHO estimates that 501,000 deaths have occurred because of unsafe injection practices [6]. Despite appropriate interventions by governments and non-governmental organizations (NGOs) working in the areas of HIV/AIDS prevention, the war against unsafe injection practices is yet to be won. In addition to the burden of morbidity and mortality, it is possible to calculate the burden of costs and years of life lost due to unsafe injection practices. There is an estimated global financial cost of US$535 million per year, and a calculated unsafe practice that is associated with 1.3 million deaths and 26 million years of life lost annually [3].

Due to over use of injections in many countries, unsafe injection practices transmit substantial proportion of blood-borne diseases [6]. Injection safety is an integral component of infection prevention and control, which is critical to healthcare services. The observation of safe injection practices will promote improved access to quality care and treatment for People Living With HIV/AIDS (PLWHA) and other blood-borne diseases [6]. Surveys have indicated that injections are the preferred methods of treatment for patients and clients [6]. Therefore, as attempts are being made to reduce the spread of HIV/AIDS and other blood-borne diseases, it is imperative that injection safety be given a priority. Although the training of health care workers on injection safety practices by John Snow Incorporated/Making Medical Injection Safer (JSI/MMIS) was done between 2004 and 2007 in all the tertiary and secondary health care facilities in Cross River State, no training was done in any of the Primary Health Care (PHC) facilities in the state because training in PHC facilities was not included in the protocol plan of John Snow Incorporated/Making Medical Injection Safety. The choice of conducting injection safety training in PHC facilities is imperative because these facilities are the entry point into Nigeria´s health care system. Hence, a pre-intervention assessment of health care providers´ knowledge and practices provides background information of the current situation on injection safety. In addition, there has been a preponderance of health education enlightenment campaigns in urban settings on safe injection practices by JSI/MMIS through radio and television advertisement as well as posters and flyers. Hence, comparing rural and urban PHC facilities will enhance the assessment of the differences, if any, in proportion of safe injection knowledge and practices especially at the pre-intervention phase. There is general lack of data on injection safety practices in Cross River State. This was a baseline study to compare knowledge and practice of safe injection practices; and factors associated with knowledge and practice of injection safety among PHC workers in Cross River State, Nigeria. The results of this study could be useful in developing public health interventions to prevent unsafe injection practices in PHC facilities in Cross River State, and, perhaps, Nigeria.

## Methods

**Study setting/area:** this study was done in PHC facilities in Cross River State, Nigeria. Cross River State is one of the six states that make up the South-South geo-political zone of Nigeria. The state has two tertiary health care institutions situated in Calabar, the state capital. These are the University of Calabar Teaching Hospital and the Federal Psychiatric Hospital. There is one secondary health care facility in each of the 18 Local Government Areas (LGA) in the state. There are many PHC facilities and privately-owned health facilities in the state.

**Study design:** this was a cross-sectional comparative study of knowledge and practices of injection safety among PHC workers in a randomly selected rural LGA and an urban LGA.

**Study population:** the study population was PHC workers in Cross River State.

**Inclusion and exclusion criteria:** all trained PHC staff in the selected PHC facilities involved in administration of injections to patients or clients were included in this study. Other Untrained PHC staff not involved in administration of injections to patients or clients were excluded from this study.

**Sample size determination:** the minimum sample size was determined using a two-proportion comparative formula. The total minimum sample size of 320 (i.e. 160 per study arm) was estimated as shown below:

$$n=\frac{0.5\times 0.5\times {\left(1.96+1.28\right)}^{2}\times 2}{{\left(0.5-0.31\right)}^{2}}$$

Where n = Minimum sample size p = proportion of unsafe injection practices from a previous study [2] = 50% (0.5) d = difference to be detected between urban and rural settings (pA - pB) = 50-31% = 19% i.e. effect size of 19% pA = proportion of unsafe injection practices from a previous study [2] (pre-intervention) = 50% = 0.5 pB = estimate of expected proportion of unsafe injection practices (pre-intervention) = 31% Z_α/2_= 95% confidence = 1.96 Z_β_= 90% power = 1.28

$$n=\frac{p(1-q){\left(Z_{\alpha /2}+Z_{\beta }\right)}^{2}\times 2}{d^{2}}$$

$$n=\frac{0.25\times {\left(3.25\right)}^{2}\times 2}{{\left(0.19\right)}^{2}}=\frac{0.25\times 10.50\times 2}{0.0361}=\frac{5.25}{0.0361}=144$$

To take care of 10% non-response: 144/0.9 = 160 Therefore, the actual minimum sample size that was selected was 160 for rural and urban groups (study) as well as rural and urban groups (control) making a total of 320.

**Sampling technique:** a multistage sampling method was used to select four LGAs for the data collection. Stage one: the National Population Commission defines a rural area as a single geographical setting or community with a population of less than 20,000 people, while an urban area is a single geographical setting or community with a population of more than 20,000 people [7]. Two LGAs were selected in each of the urban and rural settings using a simple random sampling technique (balloting). Calabar South and Yakurr were selected from the urban LGAs while Akpabuyo and Biase LGAs represented the rural LGAs. Stage two: a lists of all the PHC facilities in each selected LGA and the nominal role for each facility were collected from the LGA PHC coordinators. There were eight (8) to ten (10) PHC facilities in the urban LGAs and 10 to 12 PHC facilities in the rural LGAs, with each LGA had an average of 85 PHC health workers. Therefore, all the health workers in each study setting were recruited; hence, all population study.

**Data collection:** four Resident doctors and six Community Health Officers in the Department of Community Medicine were trained on the principles and practices of injection safety and on administration of questionnaire (Annex 1). The Fiji National Injection Safety data instrument [8], was adapted and used for the data collection. The questionnaire was pre-tested in two PHC facilities in another LGA not selected for this study. This was to see if the questions were well structured and to estimate average duration of administration of the questionnaire. Minor corrections observed were incorporated into the questionnaires for final data collection. Data were collected on respondents´ socio-demographic status, knowledge of injection safety, practice of injection safety, factors that influence unsafe injection practices. A checklist was used to observe the practice of injection safety in the health care facility.

**Data management and statistical analyses:** variables for knowledge and practices of injection safety among the respondents were recorded to enable easy analysis and interpretation of results.

**Measurement of outcome variables:** in this study, scores were contextually assigned for the purposes of measuring knowledge and practice of injection safety as shown below:

**Knowledge of injection safety:** respondents who ticked “Yes” to knowledge of injection safety were categorized as having accurate knowledge and were scored 1, while those who ticked “No” knowledge of injection safety were categorized as having inaccurate knowledge and were scored 0. Level at which a safety box should be filled before disposal: a score of 1 was assigned to respondents who ticked “¾”, a score of 0 was assigned to respondents who ticked either “½” or “Full”. Three diseases that can be transmitted by unsafe injection practices: this was recorded; any correct answer had a score of 1, if all three answers were correct the respondent scored 3, if all three answers were wrong, the respondent scored 0. The correct responses include: HIV, hepatitis B, hepatitis C, viral hemorrhagic fever, malaria and tetanus. Maximum composite score for knowledge of injection safety was 5, while the least score for knowledge of injection safety was 0. Therefore, respondents with scores <3 and ≥3 were categorized as having poor and good knowledge of injection safety respectively. Knowledge of health risks associated with unsafe injection: this was recorded; respondents were expected to supply three answers. If two or three answers were correct, this was categorized as good knowledge. If one answer was correct or all answers were wrong, this was categorized as poor knowledge.

**Practice of injection safety:** patients or clients provide their own injection equipment for therapeutic or preventive services: a score of 1 was assigned to respondents who ticked “Never”, a score of 0 was assigned to those who ticked “Sometimes”, and those who ticked “Always”. Use of the recommended diluents to reconstitute drugs and vaccines: a score of 1 was assigned to respondents who choose “Yes”, while a score of 0 was assigned to respondents who choose “No”. Re-use of syringes for drug and vaccines withdrawal: a score of 0 was assigned to respondents who ticked “Yes”, while a score of 1 was assigned to respondents who ticked “No”. Item used in collecting injection waste in health facilities; after injection administration, what was done to the needle and syringe: The answers were re-coded, only one answer was required. A correct answer was scored 1 and a wrong answer was scored 0. The correct responses include: safety box and improvised plastic containers.

Maximum observed composite score for practices of injection safety was 16 while the least score for practices of injection safety was 7. Therefore, respondents with scores <13 and ≥13 (these scores were contextually assigned) were categorized as having poor and good practices of injection safety respectively. (N/B: maximum expected composite score for practices of injection safety was 33 while the least score was 0. However, in this study the maximum observed composite score was preferred and used in the analysis for practices of injection safety). Practice of one of the “eleven rights” (“right drug”) A score of 1 was assigned to respondents who ticked “Yes”, while a score of 0 was assigned to respondents who ticked “No”. For the structured observation checklist, the correct response for majority of the questions was “Yes” with an assigned score of 1, a “No” response scored 0, except for questions: 9, 11, 12, 23, 26, 28, 29, 30 - 35 where the correct response was “No” with an assigned score of 1, a “Yes” response scored 0. For question 10, availability of syringes and needles like vanish point retractable or auto-disable syringes and needles, a score of 1 was assigned to either of the two, while a score of 0 was assigned to disposable (kojak) syringes and needles where available. Maximum composite score for injection administration procedures at health facility was 34 while the least score was 0. Therefore, respondents with scores <17 and ≥17 (these scores were contextually assigned) were categorized as having poor and good injection administration procedures at health facility respectively.

**Inferential analysis:** age of the respondents was reported in mean and standard deviation. Relative frequency was used to describe categorized age and other demographic information of the respondents in the study settings. Level of statistical significance was set at 5% significance level (p<0.05) in the bivariate analyses. Student t-test was used for testing the difference in the mean age of the respondents in the urban and rural settings. Chi square test was used to test differences between respondents´ age category, cadre of health workers, years of experience and sex in the urban and rural settings. Fisher´s exact test was used to test differences between respondents´ marital status and ethnicity in the urban and rural settings where the expected cell count was less than five were used for analysis. The difference between knowledge and practice of injection safety in the urban and rural settings was tested using Chi square. Variables found to be statistically significant with injection safety knowledge and practices in the univariate logistic regression analysis were used to model the multivariate logistic regression analysis which was used to adjust for potential confounders at 5% level of significance in order to accurately determine predictors of injection safety knowledge and practices. Statistical significance in the regression analysis was defined as the 95% confidence interval value excluding the null value of 1.STATATM version 14.0 was used for the data analysis.

Ethical clearance was obtained from Research Ethics Committee, Centre for Clinical Governance, Research and Training, Ministry of Health, Calabar, Cross River State [(CRS/MH/CGS&E-H/018/vol./11). A written permission was obtained from the PHC coordinators of the study LGAs where the aims and objectives of the study were explained to them. Informed consent and cooperation was also obtained from the participants before he/she was included in this study. The objectives of the study were explained to them and they were assured of confidentiality of their information. The participants were given the option to opt out of the study if they so wished and that their refusal to participate will not attract any punishment or denial of benefits due them.

## Results

Table 1 shows the socio-demographic characteristics of the respondents by urban vs. rural location. The mean age of the respondents in the urban setting was 39 ± 7.2 years, while that of the rural location was 41 ± 6.9 years and the difference was statistically significant (p = 0.015). There were no statistically significant differences between the respondents in the urban and rural location with respect to sex (P=0.218), marital status (P=0.301), ethnicity (P=0.110), cadre of health worker (P=0.18), and years of experience on the job (P=0.135). Table 2 shows the comparison of total composite knowledge of safe injection among respondents in the urban and rural LGAs at baseline. Overall, in the urban group, 58.8% of the respondents had good knowledge of injection safety, compared to 55% of the respondents in the rural group who also had good knowledge of injection safety. However the observed difference was not statistically significant (p = 0.499). Table 2 also shows the comparison of total composite practice of safe injection among respondents in the urban and rural LGAs at baseline. Overall, in the urban group, 33.1% of the respondents had good practice of injection safety compared to 34.4% of the respondents in the rural group who also had good practice of injection safety. However the observed difference was not statistically significant (p = 0.813). Table 3 shows the logistic regression analysis of factors associated with knowledge of safe injection at baseline. Only cadre of staff was significant in the univariate (unadjusted) analysis. The senior staff had almost a three-fold increased odds of having good knowledge of injection safety compared to the junior staff [OR=2.79 (95% CI: 1.36, 5.74)]. Table 4 also shows the logistic regression analysis of factors associated with practice of safe injection at baseline. In the multivariate (adjusted) analysis, only cadre of staff was significantly associated with practice of safe injection. The senior staff had a two-fold increased odds of having a good injection safety practice compared to the junior staff [OR=2.21 (95% CI: 1.28, 3.84)].

**Table 1:** socio-demographic characteristics of the respondents (urban versus rural)

Variable	Urban (n = 160)	Rural (n = 160)	Test of statistical significance	p-value of difference
	n (%)	n (%)		
Age group (years)			χ2	0.148
20 - 29	14 (8.8)	13 (8.1)		
30 - 39	66 (41.2)	51 (31.9)		
40 - 49	67 (41.9)	80 (50.0)		
≥50	13 (8.1)	16 (10.0)		
**Mean age**	39 ± 7.2	41 ± 6.9	Student t-test	0.015
**Cadre of health worker**			χ2	0.181
Senior	31 (19.4)	41 (25.6)		
Junior	129 (80.6)	119 (74.4)		
**Years of experience**			χ2	0.135
< 15	68 (42.5)	55 (34.4)		
≥ 15	92 (57.5)	105 (65.6)		
**Marital status**			Fisher test	0.301
Married	138 (86.3)	128 (80.0)		
Single	20 (12.5)	29 (18.1)		
Widowed	2 (1.2)	3 (1.9)		
Ethnicity			Fisher test	0.110
Efik	62 (38.8)	72 (45.0)?		
Ekoi	97 (60.6)	83 (51.9)		
Others	1 (0.6)	5 (3.1)		
Sex			χ2	0.218
Male	21 (13.1)	29 (18.1)		
Female	139 (86.9)	131 (81.9)		

**Table 2:** total composite knowledge and practice of safe injection among respondents

Variable	Urban (n = 160)	Rural (n = 160)	p-value of difference
	n (%)	n (%)	
**Composite knowledge**			0.499
Good	94 (58.8)	88 (55.0)	
Poor	66 (41.2)	72 (45.0)	
**Composite practice**			0.813
Good	53 (33.1)	55 (34.4)	
Poor	107 (66.9)	105 (65.6)	

**Table 3:** logistic regression analysis of factors associated with knowledge of safe injection at baseline

Variable	Knowledge of injection safety (N=320)	
	Unadjusted OR (95 CI)	Adjusted OR (95 CI)
**Age group (years)**		
20 - 29	1	
30 - 39	1.13 (0.46-2.74)	
40 - 49	1.80 (0.74-4.38)	
50 - 60	0.95 (0.31-2.88)	
**Sex**		
Female	1	
Male	1.85 (0.86-3.99)	
**Marital status**		
Married	1	
Single	0.59 (0.31-1.13)	
Widow/widower	1.37 (0.15-12.5)	
**Cadre of staff**		
Junior	1	
Senior	2.79 (1.36-5.74)	
**Years of experience**		
0 - 14	1	
≥ 15	1.18 (0.72-1.96)	

NB: education and religion had perfect prediction because all study participants had tertiary education and were Christians, respectively Multivariate (adjusted) regression analysis was not done because only cadre of staff was statistically significant in the univariate (unadjusted) analysis

**Table 4:** logistic regression analysis of factors associated with practice of safe injection at baseline

Variable	Safe injection practice (N=320)	
	Unadjusted OR (95 CI)	Adjusted OR (95 CI)
**Age group (years)**		
20 - 29	1	
30 - 39	1.86 (0.80-4.33)	
40 - 49	0.97 (0.43-2.20)	
50 - 60	0.66 (0.23-1.89)	
**Sex**		
Female	1	
Male	1.11 (0.60-2.03)	
**Marital status**		
Married	1	
Single	0.58 (0.32-1.08)	
Widow/widower	0.48 (0.09-2.91)	
**Cadre of staff**		
Junior	1	1
Senior	2.44 (1.43-4.19)	2.21 (1.28-3.84)
**Years of experience**		
0 - 14	1	1
≥ 15	1.74 (1.10-2.77)	1.51 (0.93-2.43)

NB: education and religion had perfect prediction because all study participants had tertiary education and were Christians, respectively

## Discussion

Injection safety practice is particularly needful in the era of HIV epidemic. There are reports that other blood-borne infections like HBV, HCV, haemorrhagic fevers and malaria are also transmissible through unsafe injection practices [6]. Some of these unsafe injection practices include: re-use of syringes and needles on multiple patients, placing used syringes and needles on surfaces prior to disposal, passing of used syringes and needles from one health worker to another before disposing it into the safety box, health workers not observing aseptic technique like hand washing hygiene and swabbing sites for injection administration, loading one syringe with multiple medications, using water or normal saline to reconstitute drugs or vaccine instead of the recommended diluents from manufacturers, over filling of safety boxes, open dumping of used syringes and needles. These unsafe injection practices cause harm to health workers, patients/clients and community. It is expected that adequate training and re-training of health workers on the knowledge and practices of injection safety, backed up with adequate supervision will promote safe injection practices in our health care facilities.

In this study, baseline data were similar between respondents in the urban LGAs and rural LGAs with respect to injection safety knowledge and practice. In this study, respondents who said their patients sometimes provided their own injection equipment for therapeutic services were more in the rural than in the urban LGAs. This is an unsafe injection practice and should not be encouraged because, the sources of these injection equipment are doubtful, mostly from scavengers and vendors who pick used syringes and needles at unsecured disposal sites, and repackage them for sale at very cheap rates to unsuspecting patients. [9] When health workers use these syringes and needles on them, the patients are prone to infection with blood-borne diseases such as HIV, HBV and HCV. The reasons adduced for this type of practice in a similar study carried out among health workers in selected health facilities in the six geopolitical regions of Nigeria [9] was that most health facilities lacked injection equipment to serve their ever increasing population, some patients no longer trust the sterile conditions of syringes and needles provided at their health facilities as well as poor requisition of injection equipment and other supplies by health facility heads. The observed practice may also be attributed to health workers pilfering the injection supplies for use in their private practice thereby rendering the supplies inadequate. The practice of the use of new syringes and needles for vaccine reconstitution among respondent in the urban and rural facilities was high (Table 2). This is a good practice that should be encouraged because this will prevent drug contamination and also prevent adverse events like injection abscesses and cellulitis to patients following contamination of injection equipment and drug. The reason for the high level of performance of this injection safety practice could probably be that the practice had been there and continually maintained by strict supervision. Logistic regression analysis revealed that only cadre of staff was significantly associated with knowledge (unadjusted model) and practice (adjusted model) of injection safety (Table 2, Table 3). This means that the senior staff were more likely to have good knowledge and practice of injection safety compared to the junior staff. This could probably be due to their years of experience, although the years of experience variable was not significant in the analysis.

## Conclusion

In view of the paucity of data on knowledge and practices of injection safety among primary health care workers in Nigeria, this study provides baseline information on safe injection practices in Cross River State. There was no significant difference at baseline between knowledge and practice of injection safety among the respondents in the urban and rural health facilities. With increased likelihood of senior staff to practice good injection procedures than their junior counterparts, senior staff should help inculcate this injection safety practices to their junior staff especially the newly employed ones to be aware and avoid any practice that will expose them to health hazards. The good knowledge on injection safety should be sustained among all PHC workers across the state by the PHC Coordinators who should conduct supervisory visits to health facilities to improve on and ensure adherence to injection safety practices. The PHC Coordinators should also liaise with their LGA Chairmen and evolve a programme of activities that will ensure periodic intensive training of PHC workers on injection safety knowledge and practices (at least twice in a year). Also, the Cross River State Director of PHC in conjunction with the State Commissioner for Health should see the urgent need to organize intensive training of PHC workers across the state on injection safety knowledge and practices as well as periodic retraining at facility level. Furthermore, research on effects of training on injection safety knowledge and practices among PHC workers is strongly recommended.

### What is known about this topic

- Unsafe injection practices are common among healthcare workers in low income countries;
- Blood borne diseases and needle stick injuries are common consequences;
- The financial cost and deaths due to unsafe injection practices annually is huge.

### What this study adds

- The knowledge of safe injection among respondents in this study was good;
- The practices of safe injection among respondents in this study was poor;
- This was just the only available baseline information among Primary Health Care (PHC) workers in Cross River State.

## Annex

**Annex 1**: study instrument questionnaire on the effect of training on knowledge and practices of injection safety: a comparative study among primary health care workers in the rural and urban local government areas in cross River State (PDF - 539 Kb)
